# The state of vascular surgery education in the United States

**DOI:** 10.3389/fsurg.2024.1409688

**Published:** 2024-05-28

**Authors:** Elizabeth Lavanga, Leana Dogbe, Jacob Soucy, Faizaan Aziz, S. Lauren Nguyen, Ahsan Zil-E-Ali, Faisal Aziz

**Affiliations:** ^1^ Department of Medical Education, Penn State College of Medicine, Hershey, PA, United States; ^2^ Department of Biology, University of Michigan, Ann Arbor, MI, United States; ^3^ Division of Vascular Surgery, Penn State Milton S. Hershey Medical Center, Heart and Vascular Institute, Hershey, PA, United States

**Keywords:** education, vascular surgery, vascular education, vascular surgeons, surgical education

## Abstract

With the growing proportion of elderly population in the US and a relatively fixed supply of well-trained vascular surgeons, there is a serious concern that we will be facing a shortage of vascular surgery workforce in the near future. One of the main reasons why there is a shortage of vascular surgeons in the US is due to the fact that many students don't get exposed to this field throughout their student lives and a recent survey of medical students from a non-urban tertiary care academic institution showed that early exposure of the medical students to the surgical careers is correlated with an increased interest in the surgical field. This review of the state of vascular surgery education in the US at the undergraduate level describes in detail the importance of an early introduction to vascular surgery in the education curricula, the current state of the education, potential avenues to improve the exposure of students to the field of vascular surgery and the importance of this effort in matching the increasing need for vascular surgeons for an aging population which is likely to require dedicated care by vascular surgeons of the future. At the present time, the two pathways by the Accreditation Council for Graduate Medical Education (ACGME) to obtain dedicated vascular surgery training in the US include either enrolling in a two year clinical fellowship after completion of general surgery training or to match in a five year vascular surgery integrated residency program after successful completion of medical degree.

## 1 Introduction

Vascular surgeons play an indispensable role in the healthcare system, managing a wide range of arterial and venous disease processes, often in complex clinical scenarios. Their pivotal role extends in providing emergent consultation for intraoperative complications such as hemorrhage and ischemia in initially unrelated to vascular conditions. This scope of practice requires mastery of treatment modalities including medications, open surgery, and endovascular techniques (1–3).

Traditionally, vascular trainees in the United States underwent a five-year general surgery residency followed by two years of vascular surgery fellowship. However, there has been a transformative shift toward the integrated training pathway. The 0 + 5 model, beginning in 2006, wherein trainees engage in five years of vascular-specific training, has allowed for a more fand efficient training approach. Between 2008 and 2021, match rates in the general surgery plus vascular fellowship pathway have remained stable at 96%–97%, attesting its appeal and reliability. Concurrently, the match rates for both vascular surgery fellowship and 0 + 5 residency training models have consistently increased with time (4).

Despite contributing to an expanding pool of vascular surgeons, both pathways fall short of addressing the workforce shortage that is expected to persist through 2,040. Current levels of access are maintained by a ratio of 1.4 vascular surgeons per 100,000 population. This equilibrium is precariously steady as the population continues to grow, and an accelerated aging public is seen, outpacing the vascular training programs output. The prevalence of peripheral arterial disease (PAD) is also increasing. These imbalances pose significant concerns to patient outcomes, especially in patients made vulnerable by inequities related to race, economic status, and geographic location (5).

This review delves into the contemporary landscape of premedical and medical vascular education and its impact on residency applications. Moreover, through the identification of current gaps in vascular education, we propose potential avenues to promote early exposure to vascular surgery, elevate student engagement, refine vascular training methodologies, and encourage interdisciplinary collaboration. These proactive measures may prove beneficial in recruiting and cultivating vascular trainees, ultimately alleviating the workforce challenges that presently confront the field of vascular surgery.

## 2 Current state of high school, undergraduate, & medical school vascular education

From high school to medical school, students encounter varying degrees of education regarding human anatomy, physiology, and pathology. A fundamental grasp of human biology, typically acquired through courses in anatomy and physiology, serves as the groundwork for understanding complex medical specialties such as vascular surgery. Surprisingly, many American youth lack exposure to these foundational courses by the culmination of their high school education and are therefore less likely to engage in higher level premedical undergraduate courses. A survey by Goltz et al. (6) showed that only 5% of first- and second-year medical students were aware of integrated vascular surgery residency programs, but many are willing to consider vascular surgery as a career if they had been exposed to this field. Similarly, Chau et al. (2) showed that surgical mentorship played a dominant role in students' choice of a surgical career and overall, burnout associated with surgical career is the most common concern for medical students.

Efforts to cultivate interest and instill passion of medicine among young individuals have been pioneered by medical professionals like Dr. Manish Mehta, a vascular surgeon based in Latham, New York. Dr. Mehta's program, V-Healthy (7), aims to educate high school students on blood pressure monitoring, subsequently leading to a research study addressing the underdiagnosis of hypertension—a significant risk factor for vascular disease—in the United States (8). These initiatives foster awareness of treatable risk factors from an early age, potentially nurturing interest in various medical fields, including vascular surgery.

As students' progress into their undergraduate studies, they encounter foundational courses in physics, chemistry, and biology, which serve to assess their aptitude for the medical field. However, institutions are increasingly emphasizing the “why” behind medicine through courses like, “The Science of Medicine” (9). Such courses delve into the historical context and future trajectory of medicine, diverging from traditional textbook-based learning to engage students through guest speakers, interactive lectures, and clinical observations. This approach enables students to explore the underlying motivations driving their pursuit of a career in medicine and exposes them to diverse patient experiences, aiding in the identification of their professional interests within the field.

Regarding the level of interest in vascular surgery have surfaced, a study published in the Journal of Vascular Surgery revealed a low level of interest among U.K. medical students, with a median score of 2.7 out of 10 (5). Factors contributing to this lack of interest include the lengthy training period (29%–58% of respondents across different surveys), family life compromises (58%), and more notably, a perceived deficiency in mentorship opportunities (43%). Survey findings from the same study indicate that medical students with an interest in vascular surgery value interactive learning methods over traditional lectures. Another survey showed that 49% of first- and second-year medical students will strongly consider vascular surgery as a career if they get exposed to the field and an additional 19% would consider vascular surgery as a career if the length of the training was reduced (6). Purported case discussions are highly regarded for their ability to simulate real-life scenarios and challenge students' critical thinking skills, with 77.6% of medical students agreeing that they preferred this method of teaching (10). However, despite the evident preference for hands-on experiences, vascular surgeons contribute minimally to medical school curricula, constituting only 0.2% of lecture hours.

The primary issue identified in vascular surgery education comes down to a lack of exposure. To address this gap, medical students require greater immersion in the field of vascular surgery, emphasizing practical experiences over classroom-based learning. Furthermore, increased involvement of vascular specialists in medical education is essential to ensure comprehensive training for the next generation of surgeons.

## 3 Future directions of medical school vascular education

Despite the evolution of vascular surgery and the significant increase in the demand for highly qualified vascular surgeons trained in complex open cases, endovascular repair and medical management, the training of medical students does not match the overall pace of disease burden on the field (11). Vascular surgery in medical schools is included in the curriculum either as a part of general surgery or cardiovascular surgery with very few lessons and lectures on some of the most common conditions vascular surgeons deal with (9). Peripheral arterial disease (PAD) has a prevalence of 3%–10% in the general population and 15%–20% in patients older than 70 years old (12). Despite its high morbidity and mortality, there is little inclusion of PAD and other major vascular pathologies into the medical school curriculum. Schwartz et al. studied the knowledge of internal medicine interns regarding the prevalence, screening, and treatment of vascular disease. Their findings demonstrated that residents scored moderately on these questions and were therefore not well equipped to handle vascular pathology due to gaps in their prior medical education (13). The Ankle-brachial Index (ABI) is a known and reliable vascular biomarker for the detection of PAD and for primary and secondary prevention of cardiovascular events. Despite its importance in the diagnosis of PAD, Wyatt et al. documented a poor level of baseline knowledge of calculation and interpretation of ABI among internal medicine residents, irrespective of the year of residency (13). These findings highlight an inadequacy of knowledge within the sphere of vascular care and requires attention as this could be related to general understanding, exposure, and interest of medical students in the patient pool largely treated by vascular surgeons.

As the demand for vascular surgeons escalates, so does the need for more vascular trainees. Go et al. in their recent study predicts a shortage of vascular surgeons through 2,040 with an estimated increase of work Relative Value Units (wRUV) by 21% in 2,030% and 7% in 2,040 by each vascular surgeon to meet the demands of society (14). This highlights the importance of increasing vascular surgery education among medical students. Studies have shown that students are more likely to be influenced to purse surgical careers when exposed earlier during their medical education (15). To promote exposure to vascular surgery and the increasing demand, the medical curriculum for medical schools should seek to involve vascular surgery as a core subject outside general surgery. As the medical school curriculum continues to highlight and focus on community care, the focus on surgery decreases even further for subspecialities like vascular surgery. Medical students should receive hands-on education regarding the appropriate steps to measure and interpret ABI and doppler results, and how to use an ultrasound to identify a thrombosed or stenosed blood vessel. Similarly teaching medical students how to auscultate for a carotid bruit and how to examine abdomen to palpate prominent aortic pulsations will go a long way to diagnose carotid stenosis and abdominal aortic aneurysms. In a study by Hirsch et al. where 83% of patients had a known diagnosis of PAD only 49% of primary care physicians were able to identify this diagnosis (10). The inability of primary care physicians to identify patients with PAD leads to delayed referrals and advanced stage of presentation to a specialist which can affect surgical outcomes.

Medical schools should seek to integrate digital tools and virtual reality into their education. Chen et al. showed that virtual reality was as efficient as other methods of tutoring in the teaching of anatomy to medical students. Furthermore, students in the virtual reality group provided more positive feedback compared to those in the cadaver and atlas groups (16). Virtual reality can also be used as an adjunct to expose students to suturing practice and the mechanics of blood flow. Medical school curriculums should also focus more on interdisciplinary collaborations. Vascular surgery is a multidisciplinary field that requires collaboration with both surgical and nonsurgical specialties. Teaching medical students how to be team players will improve patient care and promote better outcomes.

A possible avenue that can be beneficial for medical students in fostering interest is student-led vascular surgery interest groups (VSIGs). These groups can provide ample opportunities for early exposure to the field, enhance delivery of education, involvement in research and can also orchestrate activities that can serve as a focal point for medical students to explore vascular surgery as potential career path. Previous studies have reported effectiveness of student-led groups in surgery with an invaluable platform for augmenting surgical education and an extent to which the students actively considered and opted for surgical residency (17–19). Moreover, VSIGs or similar groups in medical school can bridge the gap between the medical students and the vascular surgeons and residents by facilitating activities, talks and educational sessions that can lead to enriching experience conducive to exploring vascular surgery as career.

## 4 Impact of medical school vascular education on vascular surgery residency application

Numerous medical students embark on their journey into medical school without a clear direction in the vast landscape of medicine. Even among those who enter with a specific specialty in mind, statistics reveal a notable 55%–80% pivot during their academic tenure (20). This underscores the profound impact that the experiences garnered throughout medical school exert on their ultimate choice of specialization. Three issues confront the future vascular workforce: attracting, recruiting, and retaining the essential workforce to meet the challenges of vascular disease now and in the future (21).The limited exposure to the vascular field in medical school contributes to the lack of interest in this surgical specialty. A survey of medical school graduates was conducted investigating this lack of exposure which found that two-thirds of students reported limited exposure to vascular surgery, with a mean exposure time of less than one week. This was contrasted with a mean exposure time of eight weeks in general surgery (22). Another survey revealed that, not only did students identify limited exposure to vascular surgery, but their encounters with the profession during clinical rotations was largely happenstance (23).

In the absence of exposure within the standard curriculum, medical students often resort to external avenues such as conferences for a glimpse into the vascular field. However, financial constraints often impede participation. Furthermore, the lack of reliable online information detailing a career in vascular surgery hampers informed decision-making among aspiring medical professionals (23). Compounding this, the shift from the traditional (5 + 2) to the integrated (0 + 5) educational pathway in vascular surgery underscores the critical need for early exposure to the profession (22).

To effectively address the shortage of interest in pursuing vascular surgery as a career and optimize recruitment efforts, it's imperative to delve into the motivations driving students and residents towards this field. Five key areas have been identified: Experiential Learning, Intellectuality, Mentorship, Professional Identity, and Emotional Intelligence (23). Crafting strategies to attract future residents through early exposure, facilitated by initiatives like the Vascular Surgery Interest Group (VSIG), mentorship programs, immersive clinical rotations, exposure to complex cases, and research opportunities, will enhance interest in a career as a vascular surgeon.

## 5 Dedicated vascular surgery fellowships after completion of general surgery training

As mentioned earlier, the ACGME offers two routes to obtain vascular surgery certification in the US. Section 4 outlines the impact of medical school vascular education on the choice of vascular surgery career among medical students. This section explores the factors affecting choice of vascular surgery subspeciality among general surgery trainees in the US. Traditionally, due to a shortage of vascular surgeons across the US, many vascular operations were performed by the general surgeons and this is the main reason why ACGME has included requirement to perform a certain number of vascular surgery operations during the general surgery training. But with passage of time, the number of vascular operations performed by the general surgeons have gradually diminished. A review of Medicare population from 1996 showed that only 10% of practicing general surgeons performed major vascular operations. The most common vascular surgery operations performed by the general surgeons included dialysis access and minor vascular surgery operations (24). It is important for the future of vascular surgery to continue to have a pipeline of vascular surgeons who are trained in dedicated two-year vascular surgery fellowships. A survey by Calligaro et al. (25) identified technical aspects of vascular surgery, role of mentors and complex decision making as the most important factors affecting the decision making of graduating general surgery chief residents to choose vascular surgery as a carrier. While the number of integrated vascular surgery residency positions has increased every year, the number of vascular surgery fellowships have remained steady over the past decade. It is important for our specialty to maintain the number of current vascular surgery fellowship positions to ensure adequate supply of vascular surgeons for the near future.

## 6 Technology in vascular surgery

Utilization of online learning platforms, also known as electronic learning (E-learning), in vascular surgical training has grown in importance since the coronavirus pandemic. E-learning has taken the form of online video-based conferences, mobile applications for troubleshooting industry products when representatives cannot be present, and both synchronous and asynchronous interactive training modules (26). Patelis et al. surveyed 856 participants from 84 countries to determine the utilization of E-learning programs during the COVID-19 pandemic. 84% of participants who had engaged in some form of electronic learning held positive attitudes towards E-learning (27). The merits of electronic forms of learning include elimination of the need for travel and time off from work for continuing education efforts. E-learning may also increase the personalization of learning by adapting to provider-needs during simulations and training sessions. Based on performance and training history, many E-learning programs can develop person-specific learning objectives, therefore tailoring the experience to the individual and cutting down on the teaching of unnecessary topics (28). However, E-learning also poses its own challenges including loss of hands-on opportunities, difficulty in obtaining protected work hours for E-learning, and social isolation which often accompanies computer-based activities (29).

One area of electronic learning which has grown exponentially in recent years is artificial intelligence (AI). It has been proposed that artificial intelligence will gain increasing influence in the workflow of surgical fields. Fischer et al. describes the effectiveness of AI in vascular surgery by describing its support in four primary domains: diagnostics, risk stratification, outcome predication, and perioperative medicine including the identification of at-risk patients, postoperative complications, and medical management needs (30, 31). Machine Learning (ML), a subset of artificial intelligence, is a tool that utilizes computer algorithms to predict outcomes via pattern recognition. Li et al. describes the suitability for vascular surgery to adopt ML as a tool for improved surgical outcomes and patient management. Li et al. states that vascular surgery is especially well-suited for ML integration based on four primary characteristics of the field: the increasing emphasis on endovascular techniques requiring careful anatomical planning, the presence of objective standards by which to identify candidate surgical patients, the significant number of comorbid risk factors that vascular patients endure, and lastly, the significant availability of vascular surgery perioperative data with which to train machine learning devices and programs (32). The use of AI in vascular surgery will not only increase the quality of patient care but may also improve the quality of physician life. Physicians will no longer need to spend extra after-hours time calculating patients' risk scores, sizing endovascular grafts, or planning postoperative medical therapies. This time may instead be spent increasing one-on-one time with one's patients, teaching trainees, or enjoying time with family and loved ones.

## 7 Summary and conclusions

The shaping of a future vascular surgeon begins long before one steps foot in the operating room. Efforts to improve student's exposure to the field, accurate understanding of the responsibilities of a vascular surgeon, knowledge of the training apparatuses, and a basic comprehension of vascular pathology may be achieved via earlier integration of anatomical courses into high school education, improved hands-on teaching of vascular disease in medical school, and cultivating interest during medical school years and during general surgery training could be some of the substantial approaches to prepare future vascular surgeons. Medical educational institutions must acknowledge the increasing shortage of vascular surgery practitioners by adopting the responsibility of expanding their scope of teaching to address the needs of the public.
